# Femoral Neck Fracture in the Setting of Plasmacytoma: A Case Report

**DOI:** 10.7759/cureus.42726

**Published:** 2023-07-31

**Authors:** Matthew McCrosson, Roshan Jacob, Jessyca Ray, Sameer Naranje

**Affiliations:** 1 Orthopaedic Surgery, University of Alabama at Birmingham, Birmingham, USA

**Keywords:** fracture, femoral neck, plasmacytoma, arthroplasty, hip

## Abstract

Solitary bone plasmacytoma (SBP) is a proliferation of monoclonal plasma cells found in a solitary osteolytic lesion. These osteolytic lesions can present as either bone pain or pathological fracture. We present this interesting case of a 63-year-old male that was found to have a plasmacytoma confined to the femoral neck following the presentation of a pathological fracture. After surgical resection and biopsy, we placed a distally fixating hemiarthroplasty. To our knowledge, there is only one other reported case of a pathological fracture of the femoral neck due to plasmacytoma.

## Introduction

Solitary bone plasmacytoma (SBP) is a malignant neoplasm characterized by monoclonal plasma cell proliferation in an osteolytic bone lesion found in less than 5% of all plasma cell disorders. It is a rare disorder, involving less than 450 new cases per year [1]. Specifically, the requirements for a solitary plasmacytoma diagnosis include the presence of a solitary lesion with no radiographic evidence of additional lesions. Furthermore, a bone marrow biopsy must demonstrate that the cells are either morphologically normal or have less than 10% plasma cell infiltration. Lastly, the patient must lack hypercalcemia, renal dysfunction, and anemia indicative of multiple myeloma [1,2].

Solitary plasmacytomas can be further divided into SBPs, as mentioned above, or solitary extramedullary plasmacytomas (SEPs) based on the origin of the lesion. SBPs usually occur in the axial skeleton, particularly the vertebrae, and skull; however, SEPs have also been found infrequently in the ribs, pelvis, femur, scapula, and clavicle [1-3].

We present an interesting case of a patient with a pathologic femoral neck fracture secondary to an SBP. While other studies have examined subtrochanteric pathologic fractures or metastatic disease with pathologic femur neck fractures, this is, to our knowledge, one of only two cases of an SBP that was discovered only after a sudden fracture of the neck of the femur.

## Case presentation

The patient is a 63-year-old male with a history of chronic obstructive pulmonary disease (former firefighter) and skin cancer status post excisional biopsy (done several years prior at an outside hospital; results unknown) that presented to the emergency department (ED) as a transfer after a ground level fall onto his left hip with a suspicious lesion on imaging. Upon arrival, the patient was hemodynamically stable, alert, and oriented to person, place, and time. The initial physical exam revealed that the patient was neurovascularly intact throughout the lower left extremity. The patient claims to have been stepping down from his porch when his left foot landed awkwardly, causing him to fall on his left side. Upon landing, he felt a “pop” and had immediate pain in the left hip. He denied preceding hip pain before the fall and contact with the ground. 

X-rays at the outside hospital (OSH) revealed a left femoral neck fracture with a possible lesion. He also underwent computed tomography (CT) of the chest, abdomen, and pelvis, along with magnetic resonance imaging (MRI) of the femur. Per the OSH records, no other suspicious lesions were found. He was transferred to our institution for further oncologic management. He has a surgical history of bilateral total knee arthroplasties, a right total shoulder arthroplasty, three left rotator cuff surgeries, and an anterior cervical discectomy with fusion (ACDF). 

**Figure 1:**
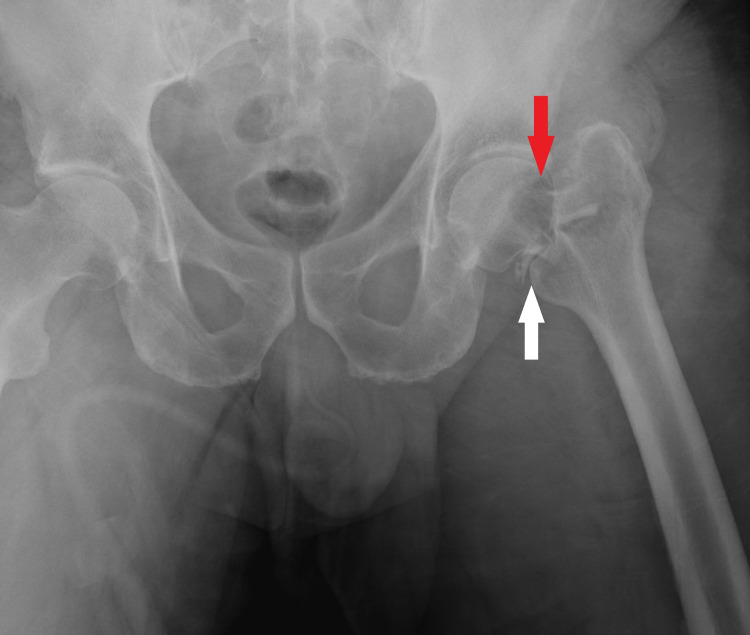
X-ray revealing comminuted left femoral neck fracture (White Arrow) with a possible suspicious lytic lesion (Red Arrow) on the femoral neck

In our institution’s ED, x-rays revealed a comminuted left femoral neck fracture with a possible suspicious lesion on the femoral neck (Figure 1). Further review of the OSH chest, abdomen, pelvis CT and femur MRI revealed no further lesions. A positron emission tomography (PET) and bone scan were considered but not performed due to the negative CT, MRI scans (besides the femoral neck lesion), and lab results. The patient had no neurologic deficits. No gross hematologic abnormalities were found on the initial complete blood count (CBC) with differential. The white blood cell (WBC) count was 9,900/ul, the red blood cell (RBC) count was 4,830/ul, the percent neutrophils were 71%, and the percent of lymphocytes was 16%. Urinalysis revealed 2+ proteins, >25 red blood cells (RBCs), and 6-10 white blood cells (WBCs). Serum calcium was at 9.1 mg/dl, creatinine was at 1.2 mg/dl, albumin was 4 g/dl, lactate dehydrogenase was 167 u/l, beta-2 microglobulin was 1.57 ug/ml, and c-reactive protein was elevated at 190.34. Serum protein electrophoresis revealed a small 0.19 gm/dl monoclonal (M) spike. There were no elevations on serum free light chain analysis, with the Kappa/Lambda ratio at 0.93, suggesting one immunoglobulin G (IgG) lambda monoclonal proliferation.

After a discussion with the orthopedic oncology faculty and with concerns about possible pathological causes, the patient agreed to an excisional biopsy followed by hemiarthroplasty. The decision was made to do the excisional biopsy during the hemiarthroplasty due to the preceding clinical workup and imaging results suggesting the lesion was an SBP rather than other neoplasms. Because of workup results, it was also determined at this time to press-fit the femoral stem with minimal reaming, a lack of other lesions on femoral MRI, good proximal femur bone quality, and approval from the department’s orthopedic oncology team.

**Figure 2:**
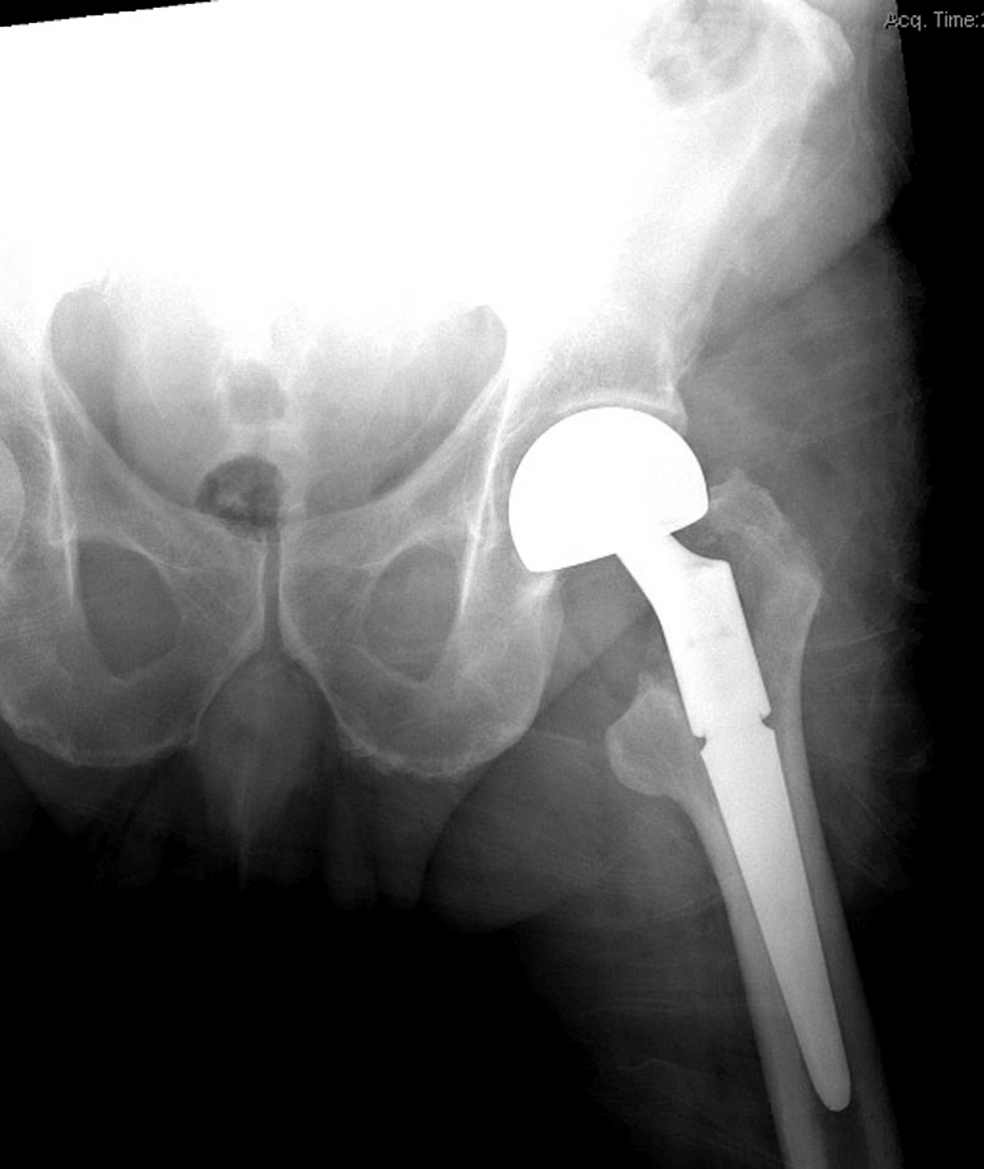
Press Fit hemiarthroplasty with a bipolar head. Initial postoperative X-ray of the left hip after distally fixating hemiarthroplasty.

The patient was prepped in a lateral decubitus position, and a classical posterior approach was performed. After capsulotomy, the comminuted femoral neck was visualized, and the femoral head was removed and sent to pathology. Additionally, several more bone and soft tissue samples were taken and sent to pathology for gross and microscopic analysis. Further exposure of the femur revealed lysis of the trochanteric ridge. Because of this, it was decided to use a femoral stem with more distal fixation, so the Revival Modular Stem from Corin was used, with the distal stem being 100 mm in length with a 20 mm diameter. The cone body was 135 degrees with a 40 mm diameter. A 40 mm Corin Revival locking screw was used to secure the implant. Following minimal reaming so that the smallest Revival stem by diameter could be used, the stem was press fit into place with good fixation, and a short Corin bipolar head was used (Figure 2). The hip was reduced, and the patient’s surgical wound was closed in standard fashion. There were no complications in the postoperative period.

**Figure 3:**
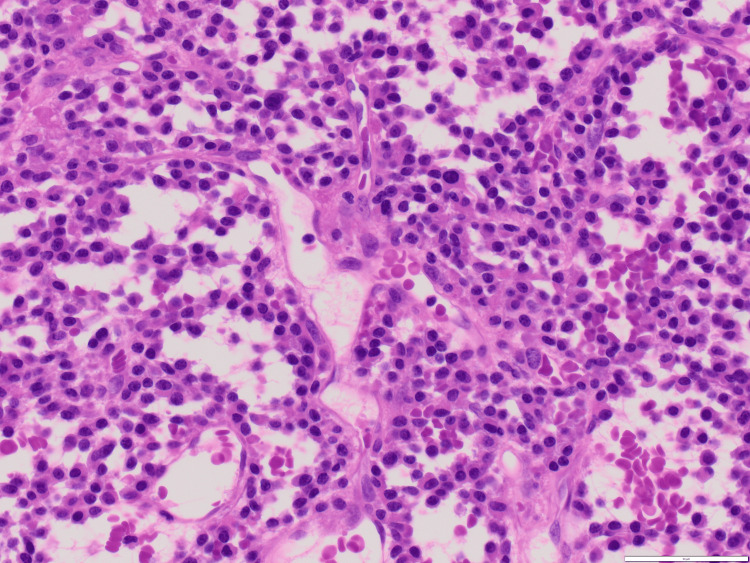
Light microscopy revealing plasma cells with large basophilic nuclei and elevated nuclei to cytoplasmic ratio. Light microscopy 40 times magnification.

**Figure 4:**
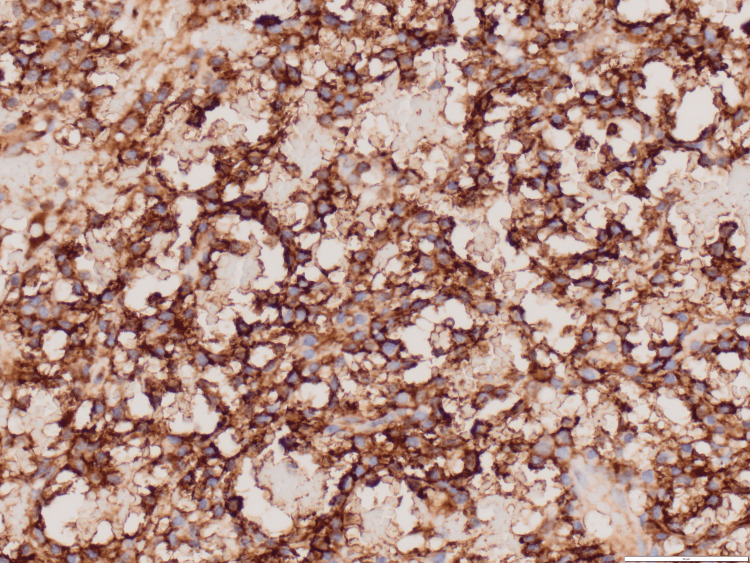
Immunohistochemistry of tissue samples positive for CD138 Light microscopy 40 times magnification.

Simple light microscopy revealed plasma cells with large basophilic nuclei and an elevated nuclei-to-cytoplasmic ratio (Figure 3). Immunohistochemistry of tissue samples was diffusely positive for CD138 (Figure 4), a cell marker for plasmocytic differentiation. Furthermore, the sample was negative for pancytokeratin (epithelial tumor marker), CD45, CD117, CD163, Sry-related HMg-Box gene (SOX) 10, avian v-ets erythroblastosis virus E26 oncogene homolog (ERG), and synaptophysin, suggestive of plasmacytoma. Following his initial postoperative visit, the patient returned to his hometown. At his 15-month postoperative follow-up, the patient reported being very satisfied with his surgery, demonstrating good hip flexion, and having no pain with log roll. He also reported that he has been following up with an orthopedic surgeon in his hometown.

## Discussion

Plasma cell dyscrasias are categorized into myeloma or plasmacytoma. Myeloma indicates a more systemic disease with indicators of advanced disease, including hypercalcemia, renal dysfunction, anemia, bone lesion (CRAB) symptoms, organomegaly, and a large M protein spike. Plasmacytomas are a localized proliferation of plasma cells, the most common of which are SBPs. SBP is diagnosed after excluding systemic myeloma and is found in a singular disease site, most commonly in red marrow-containing bones such as vertebrae, the femur, pelvis, and ribs [4,5]. Recommended diagnostic criteria for SBP include a solitary bone lesion with the absence of plasma cell proliferation on bone marrow biopsy [1]. Minor criteria typically include an absence of anemia, hypercalcemia, or renal involvement. CT scan and MRI will typically show a sharply demarcated osteolytic lesion with enhancement on contrast CT and no surrounding reactive sclerosis [5]. A biopsy of the lesion showing clonal plasma cell infiltration is required to confirm the diagnosis of SBP [6]. The patient presented in the present report demonstrated a single plasmacytoma lesion confirmed after full body CT and presented with a pathological femoral neck fracture. He did not show any hematologic abnormalities or signs of systemic myeloma.

After a review of the literature, we discovered only one other reported case of a plasmacytoma confined to the femoral neck, which also involved a paraneoplastic syndrome consisting of polyneuropathy, organomegaly, endocrinopathy, monoclonal gammopathy, and skin changes (POEMS) [7]. POEMS is a multisystem disease not considered to be myeloma [7]. Our patient, however, did not meet these criteria for POEMS and had only a pathological fracture at the time of presentation. Several studies have shown plasmacytomas or metastatic cancers in the subtrochanteric region extending into the femoral neck, which can be treated with a prosthesis, intramedullary nailing, or internal fixation [8,9]. However, lesions confined to the femoral neck typically only have one treatment option, which must be distinguished from a typical osteoporotic femoral neck fracture.

In the presented case, the patient was found to have an osteolytic lesion on the femoral neck that led to a pathological fracture that would not have fared well with standard reduction via a cephalomedulary nail and lag screw, as fixation rate failures have been reported as high as 22% in these type of fractures [9]. Although it is well documented that pathological fractures of the femoral neck are best treated with hemiarthroplasty, these can also carry a fixation failure rate of up to 10% [9,10].

Advances in femoral stem design and modular capabilities have vastly improved the surgeon’s ability to choose the type and location of fixation in the femoral canal. In this case, a modular stem with a distal fixation component was chosen to avoid additional stress on an already compromised bone stock at the base of the femoral neck. Several studies have shown that periprosthetic fracture and dislocation or loosening are the leading reasons for reoperations in pathological proximal femur fractures treated by hemiarthroplasty [8,10-12]. However, distally fixating stems have been shown to have excellent results in the medium to long term [13]. Further investigation is required to determine the effectiveness of these distally fixating stems in the setting of a pathological fracture of the femoral neck.

One area in the initial workup of this patient we would alter in the future is the lack of a PET scan to assist in ruling out further lesions and metastatic disease. While we utilized a chest, abdomen, and pelvis CT scan and a femoral MRI, we realized this was inadequate in giving us the best chance of identifying any other potential lesions, as indicated in previous studies [14,15].

To our knowledge, this is the second reported case of a plasmacytoma entirely confined to the femoral neck leading to pathological fracture and the only case in which the systemic disease, POEMS, was not associated. These lesions differ from proximal femur lesions involving the trochanteric region regarding prevalence and treatment options. Early diagnosis and treatment are critical in managing these lesions to prevent disease progression to multiple myeloma.

## Conclusions

We report a case of a 63-year-old male that presented with a pathological femoral neck fracture due to a localized plasmacytoma on the femoral neck with no systemic symptoms. The patient was treated with a distally fixating hemiarthroplasty after resection of the lesion. These osteolytic lesions unpredictably compromise bone integrity, and unlike osteoporotic femoral neck fractures, we recommend a distally fixating hemiarthroplasty in treating osteolytic bone lesions of the femoral neck. In the future, further investigation into the treatment options for SBP of the femoral neck is warranted and should address topics such as surgical approach, press-fitting versus cemented stems, and patient outcomes.
